# Antibiotics in Early Life Alter the Gut Microbiome and Increase Disease Incidence in a Spontaneous Mouse Model of Autoimmune Insulin-Dependent Diabetes

**DOI:** 10.1371/journal.pone.0125448

**Published:** 2015-05-13

**Authors:** Sophie Candon, Alicia Perez-Arroyo, Cindy Marquet, Fabrice Valette, Anne-Perrine Foray, Benjamin Pelletier, Cristian Milani, Marco Ventura, Jean-François Bach, Lucienne Chatenoud

**Affiliations:** 1 Université Paris Descartes, Sorbonne Paris Cité, F-75475, Paris, France; 2 INSERM U1151, Hôpital Necker-Enfants Malades, Paris, France; 3 CNRS UMR 8253, Hôpital Necker-Enfants Malades, Paris, France; 4 Laboratory of Probiogenomics, Department of Life Sciences, University of Parma, Parma, Italy; Instutite of Agrochemistry and Food Technology, SPAIN

## Abstract

Insulin-dependent or type 1 diabetes is a prototypic autoimmune disease whose incidence steadily increased over the past decades in industrialized countries. Recent evidence suggests the importance of the gut microbiota to explain this trend. Here, non-obese diabetic (NOD) mice that spontaneously develop autoimmune type 1 diabetes were treated with different antibiotics to explore the influence of a targeted intestinal dysbiosis in the progression of the disease. A mixture of wide spectrum antibiotics (i.e. streptomycin, colistin and ampicillin) or vancomycin alone were administered orally from the moment of conception, treating breeding pairs, and during the postnatal and adult life until the end of follow-up at 40 weeks. Diabetes incidence significantly and similarly increased in male mice following treatment with these two antibiotic regimens. In NOD females a slight yet not significant trend towards an increase in disease incidence was observed. Changes in gut microbiota composition were assessed by sequencing the V3 region of bacterial 16S rRNA genes. Administration of the antibiotic mixture resulted in near complete ablation of the gut microbiota. Vancomycin treatment led to increased *Escherichia*, *Lactobacillus* and *Sutterella* genera and decreased members of the *Clostridiales* order and *Lachnospiraceae*, *Prevotellaceae* and *Rikenellaceae* families, as compared to control mice. Massive elimination of IL-17-producing cells, both CD4+TCRαβ+ and TCRγδ+ T cells was observed in the lamina propria of the ileum and the colon of vancomycin-treated mice. These results show that a directed even partial ablation of the gut microbiota, as induced by vancomycin, significantly increases type 1 diabetes incidence in male NOD mice thus prompting for caution in the use of antibiotics in pregnant women and newborns.

## Introduction

The concept emerged over the past 20 years that the significant increase in the incidence of autoimmune and allergic diseases observed in developed countries could be secondary to the decrease in the surrounding infectious burden [1]. More recently it appeared that the gut microbiota composition, which itself closely depends on the environment, could play a major role in this trend [2,3]. Thus, interesting data showed a reduction in the diversity of the intestinal microbiota in various autoimmune and allergic diseases in humans [3–6]. To establish a causal relationship experimental models were used, mostly in allergy, where modifications in the diversity of the gut microbiota induced by different antibiotic treatments showed exacerbation of allergic asthma [7–9]. Also, short intensive antibiotic treatment of adult mice increased respiratory allergy with the concomitant occurrence of gastritis [10].

Here, we focused on autoimmunity using the non-obese diabetic (NOD) mouse model that spontaneously develops insulin-dependent diabetes (T1D) due to the selective destruction of pancreatic β-cells by autoreactive CD4 and CD8 lymphocytes [11,12].

It is now well established that occurrence of T1D in NOD mice depends on the sanitary conditions in which mice are housed [1]. Mice bred in a Specific Pathogen Free (SPF) environment are more prone to develop diabetes than mice bred in a conventional animal facility [1]. When contaminated by different bacteria, viruses or parasites, SPF NOD mice do not develop T1D [13–15]. A germ free (GF) or axenic status established under stringent conditions (i.e. mice born by caesarean section and housed in isolators) increases T1D incidence [16–18], the increase being more evident in GF males than females [16,19].

Some authors have proposed that the composition of the gut microbiota could impact on the frequency of T1D just as it does modify the occurrence of inflammatory bowel or allergic diseases. First, in patients, a correlation between the reduction in the diversity of the intestinal microbiota and the occurrence of T1D or anti-islet-cell autoimmunity has been reported [3,5,6,20]. Secondly, in SPF NOD mice and spontaneously disease-prone Bio-Breeding rats (BB-DP), disease is prevented by administration of probiotics [16,21–23]. Finally, lowering the pH of drinking water concomitantly alters the composition of gut microbiota and reduces T1D incidence in NOD mice [24].

In the present work we treated NOD mice with antibiotics that when administered to mothers early in pregnancy and to newborns until the occurrence of diabetes, induced an increase in disease incidence. This result was obtained with both a mixture of wide spectrum antibiotics (i.e. streptomycin, colistin and ampicillin) that virtually destroys the entire intestinal microbiota and, interestingly enough, also with vancomycin, an antibiotic which fundamentally alters the microbiota diversity but does not eliminate all commensals.

This increase in the occurrence of autoimmune diabetes was mainly observed in male mice, raising the question of the mechanisms underlying the difference in disease incidence between males and females [16,19,25,26].

## Materials and Methods

### Mice and antibiotic treatment

Male and female NOD mice (*H2* ^*g7*^) were bred and housed under specific pathogen-free (SPF) conditions in our animal facility.

NOD breeding pairs (n = 35) were given vancomycin (0.2mg/ml) or an antibiotic mixture composed of streptomycin (5mg/ml), colistin (1mg/ml) and ampicillin (1mg/ml) (Strep-Col-Amp) or no antibiotic (control group) in drinking water. The doses were selected based on what is described in the literature [7,27]. All antibiotics were purchased from Sigma-Aldrich (Saint-Quentin Fallavier, France). Diabetes was monitored by testing for glycosuria and glycemia using colorimetric strips. Mice were monitored once a week for glycosuria, using Accu-Check Keto-Diabur-Test 5000 (Roche, Boulogne-Billancourt, France). Diabetes was confirmed by testing fasting glycemia (>250mg/dl; Hemoglukotest and Reflolux F; Boehringer-Mannheim). This study was carried out in strict accordance with the recommendations of European Directives (86/609/EEC) and institutional guidelines (INSERM, Faculté Paris Descartes). The protocol was approved by the Ethic Committee of Paris Descartes University (registered number: P2.CK.153.10). The animal facility has an agreement delivered by the Prefecture de Police of Paris, France (registered number: C 75-15-15).

### Microbiota Analysis

Feces samples were collected from 8 week-old mice in each antibiotic-treated group (vancomycin n = 6, Strep-Col-Amp n = 7). Control samples were from 13 untreated 8-week-old mice. Bacterial DNA was extracted with the FastDNA SPIN kit, using a FastPrep instrument (MP Biomedicals, Illkirch, France). 16S rRNA amplifications were performed as previously described [28]. Briefly, the PCR products derived from amplification of 16S rRNA sequences were purified by electrophoretic separation on a 2% agarose gel and the use of a Wizard SV Gen PCR Clean-Up System (Promega, Charbonnières, France), followed by a further purification step involving the Agencourt AMPure XP DNA purification beads (Beckman Coulter Genomics GmbH, Bernried, Germany) in order to remove primer dimers. DNA concentration of the amplified sequence library was estimated through the Multitape Station (Agilent Technologies, Santa Clara, CA, USA). From the concentration and the average size of each amplicon library, the amount of DNA fragments per microliter was calculated and libraries for each run were diluted to 3E9 DNA molecules prior to clonal amplification. Emulsion PCR was carried out using the Ion OneTouch 200 Template Kit v2 DL (Life Technologies, Monza, Italy) according to the manufacturer’s instructions. Sequencing of the amplicon libraries was carried out on a 316 chip using the Ion Torrent PGM system and employing the Ion Sequencing 200 kit (Life Technologies) according to the supplier’s instructions. DNA sequencing was performed at GenProbio ltd (Parma, Italy). After sequencing, the individual sequence reads were filtered by the PGM software to remove low quality and polyclonal sequences. Sequences matching the PGM 3′ adaptor were also automatically trimmed. All PGM quality-approved, trimmed and filtered data were exported as bam files. The bam files were processed using QIIME [29]. According to what is previously published [28], quality control of sequencing reads retained sequences with a length between 140 and 400 bp, homopolymers <8 bp and mean sequence quality score >25. Additionally, sequences were truncated at the first base if a low quality rolling 10 bp window was found and sequences with mismatched primers were omitted. Lastly, primers were removed before analysis with QIIME [28]. The data set is available in the Sequence Read Archive (SRA) of NCBI, accession n° PRJNA254809.

### Intestinal cells isolation and flow cytometry

Mononuclear cells from the lamina propria of the ileum and colon were isolated from 10-week old mice. Intestines were dissected, washed with cold phosphate buffer saline (PBS), Peyer's patches were removed. One cm pieces were cut and incubated in 50ml PBS-Ca/Mg free containing 30mM EDTA for 30min at 4°C. Fragments were washed with intense shaking to detach intraepithelial cells and passed through a 70μm cell strainer. Remaining fragments were cut into 2–3 mm pieces and placed in 10ml fresh digestion solution (RPMI 1640 supplemented with 10% FCS, 1mg/ml collagenase D and 1U/ml DNase I; all from Sigma-Aldrich, Lyon, France) during 10 min at 37°C. The digestion was repeated 3 to 5 times; the suspension was passed through a 70μm cell strainer, washed in RPMI 1640 and resuspended in 5ml of 40% Percoll and overlaid on 5ml of 80% Percoll (Sigma-Aldrich). Lymphocytes were collected at the interface following centrifugation for 15min at 2.800 rpm at room temperature and resuspended in culture medium.

Isolated cells were stained for the membrane markers CD45.1, CD4, CD3, CD8, TCRαβ, TCRγδ and CD25 using antibodies from BD Biosciences (Le Pont de Claix, France). Intracellular staining of FoxP3 was performed after membrane permeabilization (eBioscience SAS, Paris, France). For cytokine production, cells were cultured for 4 h at 37°C in 96-well plates in RPMI-10% FCS (Invitrogen, Cergy Pontoise, France) containing 50ng/ml of phorbol 12-myristate 13-acetate (PMA) and 500ng/ml of ionomycin. Brefeldin A (10 ng/ml) was then added in all wells for 2 additional hours. For intracellular staining, cells were washed once and fixed/permeabilized at 4°C using the Fixation/Permeabilization kit (eBioscience) according to the manufacturer’s instructions and stained with anti-IL-17 and anti-IFNγ antibodies (BD Biosciences). Isotype controls were used for all flow cytometry stainings.

Stained cells were analyzed with a FACS CantoII cytometer (BD Biosciences) and the FlowJo software (FlowJo, Ashland, OR, USA).

### Gene expression analysis

Ileum and colon, Peyer’s patches and mesenteric lymph nodes were snap frozen and total RNA was purified using RNAeasy mini kit (Qiagen, Courtaboeuf, France). cDNA conversion was performed using ImPromII Reverse Transcription System (Promega). Real-time PCR amplification of *Il17a*, *Ifng*, *Tgfb*, *Foxp3*, *Il10*, *Cd3* and *Hprt* was performed using Pre-developed Taqman gene expression assay reagents (*Il17a* Mm00439618_m1, *Ifng* Mm01168134_m1, *Tgfb* Mm01178820_m1, *Foxp3* Mm00475162, *Il10* Mm00439614_m1, *Cd3* Mm00599683_m1, *Hprt* Mm01545399_m1) and Taqman Universal master mix (Life Technologies SAS, Saint Aubin, France) according to manufacturer’s instructions on an ABI PRISM 7900HT Sequence detection system (Life Technologies SAS). The 2-ΔΔCt method was used for relative quantification of gene expression. Individual samples were tested in duplicate and mean values were deduced. mRNA expression of *Il17a*, *Ifng*, and *Foxp3* was normalized to *Cd3* expression levels while *Tgfb* and *Il10* expression was normalized to the housekeeping gene *Hprt*.

### Histological analysis

Pancreata were collected, fixed in 4% formaldehyde and paraffin-embedded. Serial 5-μm sections were stained with hematoxylin and eosin. Mononuclear cell infiltration was scored by counting about 80–100 independent islets/recovered pancreas and distinguishing three distinct patterns that were: 1) Intact islets: islets showing a normal morphology and total absence of infiltrating mononuclear cells, 2) Peri-insulitis: islets showing a generally preserved morphology but presenting a significant number of infiltrating mononuclear cells that remained confined to the periphery of the islets and 3) Destructive insulitis: islets showing a disrupted morphology and a significant number of invading mononuclear cells.

### Statistical analysis

Cumulative diabetes incidence was evaluated by actuarial survival curves using the Kaplan-Meier estimation. Differences in the incidence curves and their statistical significance were calculated using the nonparametric log-rank test (appropriate to use when the data are adequately skewed and censored).

For gene expression, cell phenotyping and cytokine analysis, 5 mice per experimental group were analyzed individually. For each parameter assessed mean values were deduced from the data recovered for the 5 individual mice tested per group. For each organ tested, each sex (males or females) and each parameter, mean values were compared between treated and control mice using the nonparametric Mann-Whitney test.

All tests were performed using Prism software (GraphPad Software, La Jolla, CA, USA).

## Results

### Antibiotic treatment increases the incidence of type 1 diabetes

**Fig 1:**
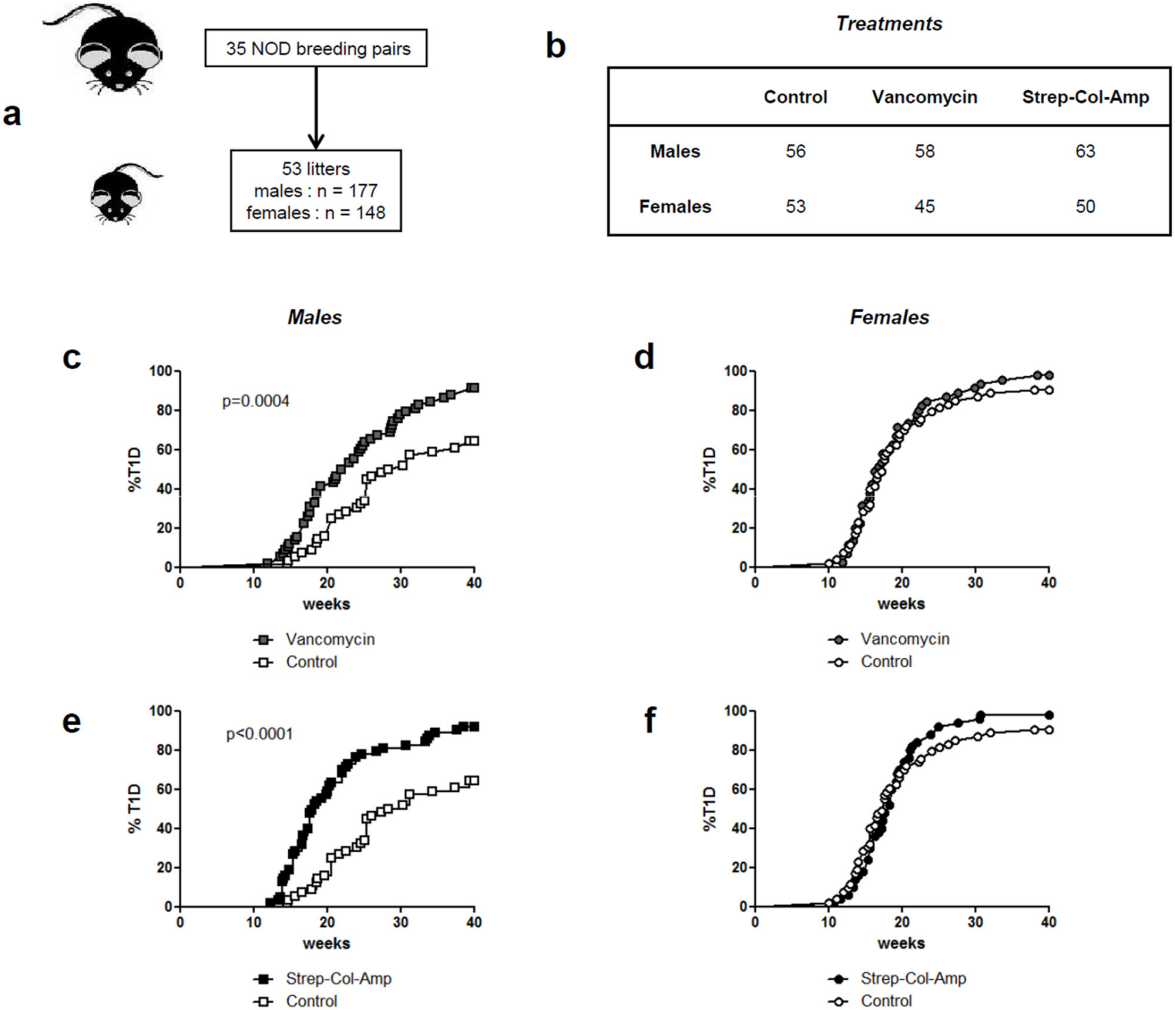
Diabetes incidence in antibiotic-treated NOD mice. The protocol used to treat NOD mice is detailed in panels **(a)** and **(b)**. Antibiotics were given in drinking water. A total of 35 breeding pairs were given either vancomycin (0.2mg/ml) or an antibiotic mixture composed of streptomycin (5mg/ml), colistin (1mg/ml) and ampicillin (1mg/ml) (Strep-Col-Amp). Controls received normal drinking water. Antibiotic treatment was started at mating and administered continuously during pregnancy and pursued for the whole duration of the experiment. Incidence of T1D was significantly increased in vancomycin-treated males **(c)** but not in females **(d)**. Similarly incidence of T1D was significantly increased in Strep-Col-Amp-treated males **(e)** but not in females **(f)**.

We exposed NOD mice to antibiotics from the moment of conception, treating breeding pairs, and during the postnatal and adult life until the end of follow-up at 40 weeks. A total of 35 breeding pairs were included that gave birth to 8–12 pups each (Fig 1a). When they were weaned at 3 weeks of age, female and male pups were separated and they were gathered with other littermates to a total of 5 individuals per cage. In the end a total of 148 females (about 30 cages) and 177 males mice (about 35 cages) were treated (Fig 1b).

We compared the effect of two protocols of antibiotic therapy. We used a wide spectrum treatment that was a mixture of streptomycin, colistin and ampicillin (Strep-Col-Amp) that targets both Gram-positive and Gram-negative bacteria [27]. This treatment has been used for the purpose of very significantly ablating the gut microbiota.

In parallel, we also applied a single antibiotic treatment using vancomycin, a glycopeptide antibiotic mostly targeting Gram-positive bacteria [7]. Both treatments were perfectly well tolerated.

The incidence of T1D in untreated NOD mice was, as regularly observed in the various reported colonies [16], higher in females (90.6%) than in males (64.3%) (p<0.0001).

Vancomycin induced a significant increase in T1D incidence in male NOD mice (cumulated incidence at 25 weeks in treated versus untreated mice: 62.1% versus 32.1%; cumulated incidence at 40 weeks: 91.4% versus 64.3% respectively, p = 0.0004) (Fig 1c). Disease onset was also significantly accelerated: the median age of diabetes onset in vancomycin-treated males was 22.4 weeks as compared to 29.4 weeks in controls, p = 0.0004. At variance with the clear-cut results observed in male mice, only a slight yet not significant trend towards an increase in disease incidence was observed in female NOD mice (cumulated incidence at 25 weeks in vancomycin-treated versus untreated mice: 84.4% versus 79.2% respectively; cumulated incidence at 40 weeks: 97.8% and 90.6% respectively, p = 0.374) (Fig 1d).

In vancomycin-treated mice and controls, islet infiltration was analyzed in pancreata recovered at 10 weeks of age from 4–5 mice. In female control mice 56.4%, 14.4% and 29.2% of islets presented no infiltration, peri-insulitis or invasive insulitis respectively (mean values from 5 individual animals analyzed). In vancomycin-treated females the proportions were 28.8%, 21.6% and 49.5% respectively. Thus, a trend towards more severe infiltration was present which, however, was not significant. In male control mice 63.9%, 22.7% and 13.4% of islets presented no infiltration, peri-insulitis or invasive insulitis respectively. In vancomycin-treated males the proportions were 33.4%, 25.6% and 41.0% respectively. Here again, a trend which did not reach statistical significance was found.

Also with the antibiotic mixture of Strep-Col-Amp a significant increase in T1D incidence was observed in treated males as compared to controls (cumulated incidence at 25 weeks: 77.8% *versus* 32.1% respectively; cumulated incidence at 40 weeks: 92.1% *versus* 64.3% respectively, p<0.0001) (Fig 1e). Disease onset was also significantly accelerated in Strep-Col-Amp-treated males *versus* controls (median age of diabetes onset 18.4 weeks in treated males as compared to 29.4 weeks in controls, p<0.0001). As observed with vancomycin, only a slight yet not significant trend towards an increase in disease incidence was observed in Strep-Col-Amp-treated female NOD mice (cumulated incidence at 25 weeks in treated versus untreated mice: 92.0% versus 79.2% respectively; cumulated incidence at 40 weeks: 98.0% and 90.6% respectively, p = 0.374) (Fig 1f).

### Impact of antibiotic treatment on the gut microbiota

Changes in gut community composition were assessed by sequencing the V3 region of bacterial 16S rRNA genes. A total of 7614709 quality reads were obtained from 36 samples (S1 Table). Importantly, samples were collected in order to obviate as much as possible cage, parent litter and age effects, that are all factors known to affect microbiota composition.

**Fig 2:**
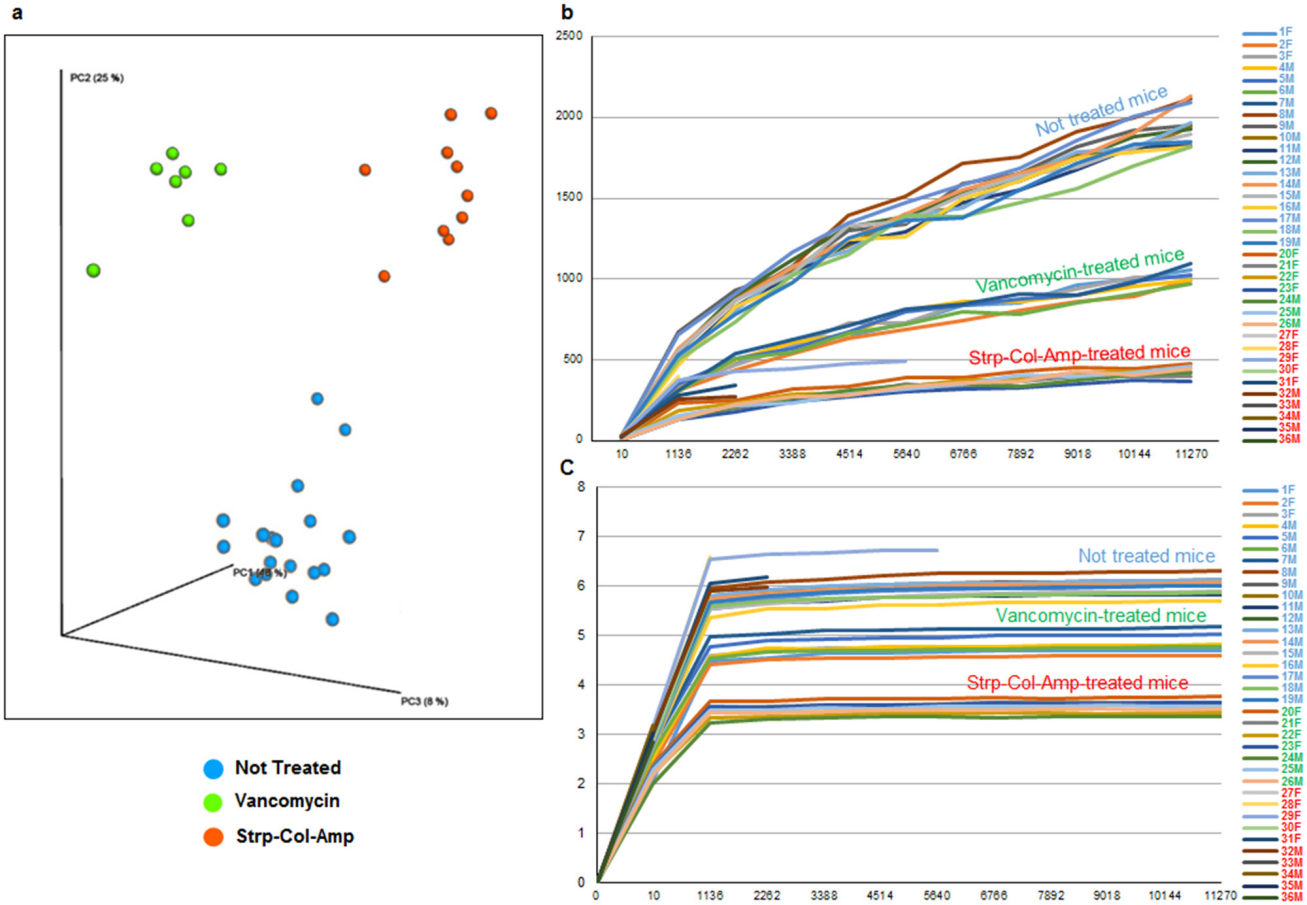
Impact of the antibiotic treatment on Alpha and Beta-diversity of mice microbiota. Panel **(a)** shows a three dimensional representation of the PCoA analysis. Panels **(b)** and **(c)** show the rarefaction curves obtained using the Chao1 and Shannon indexes, respectively.

Principle coordinate analysis (PCoA) showed that antibiotic treatment had a large impact on community composition, (Fig 2a). In fact, 48% of the variability was explained on axis PC1 and 25% of the variability was explained on axis PC2, while the analyzed samples clearly clustered separately depending on the treatment received. This emphasizes the large shift in community composition following the administration of the Strp-Col-Amp treatment compared to vancomycin treatment and compared to controls (Fig 2a). In addition, diversity was significantly lower in mice treated with antibiotics, as showed by the rarefaction curves based on the Chao1 and Shannon indexes (Fig 2b and 2c). Strikingly, the average Chao1 index value dropped from 1605 in control mice to 428 in those treated with vancomycin treatment and to189 in those subjected to Strp-Col-Amp treatment.

In mice having received the antibiotic mixture, nearly all bacterial communities had disappeared. This is clearly supported by the reduced amount of 16S rRNA amplicon sequenced and an increase in aspecific amplicons that were removed in the filtering process (S1 Table).

**Fig 3:**
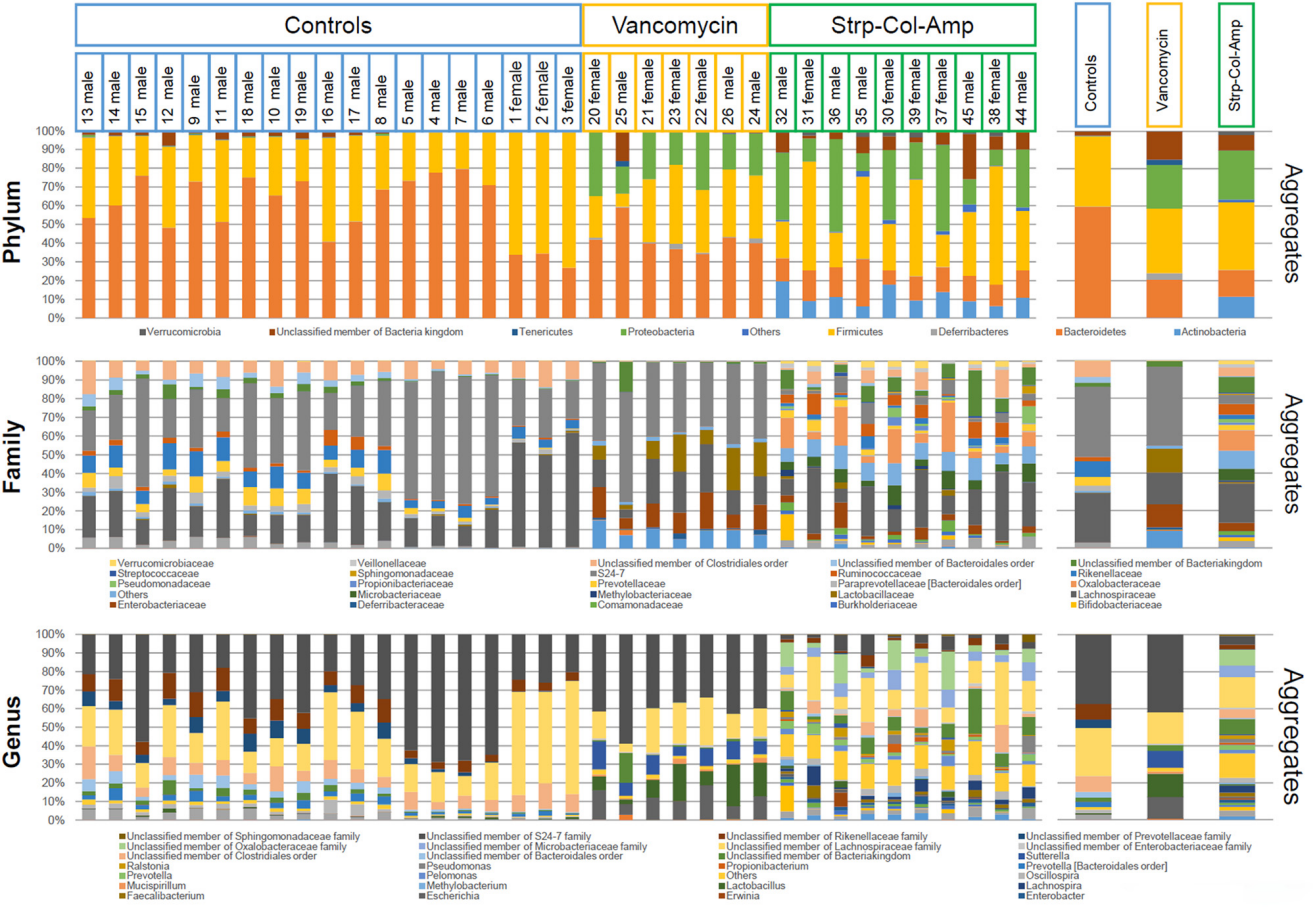
Microbiota composition of control and treated mice at phylum, family and genus levels. The bar plots show the microbiota composition at phylum, family and genus levels of the 36 mice fecal samples analyzed by 16S rRNA profiling, divided by treatment. On the right side three bar plots show the aggregate (average) bacterial composition of the three treatment conditions. Only taxa with relative abundance > 2% in at least one sample are shown.

The 16S rRNA sequences were taxonomically identified at the phylum, as well as family and genus levels using the Qiime software suite [29], based on the GreenGenes database and the RDP classifier. The 16S rRNA genes and their frequencies classified at these various taxonomic levels in feces from control or antibiotic-treated animals, further emphasize how significantly the bacterial community in the gut changed after antibiotic treatment, irrespective of sex (Fig 3). As expected, Strp-Col-Amp administration resulted in the near complete ablation of the gut microbiota represented in control mice (Fig 3).

**Fig 4:**
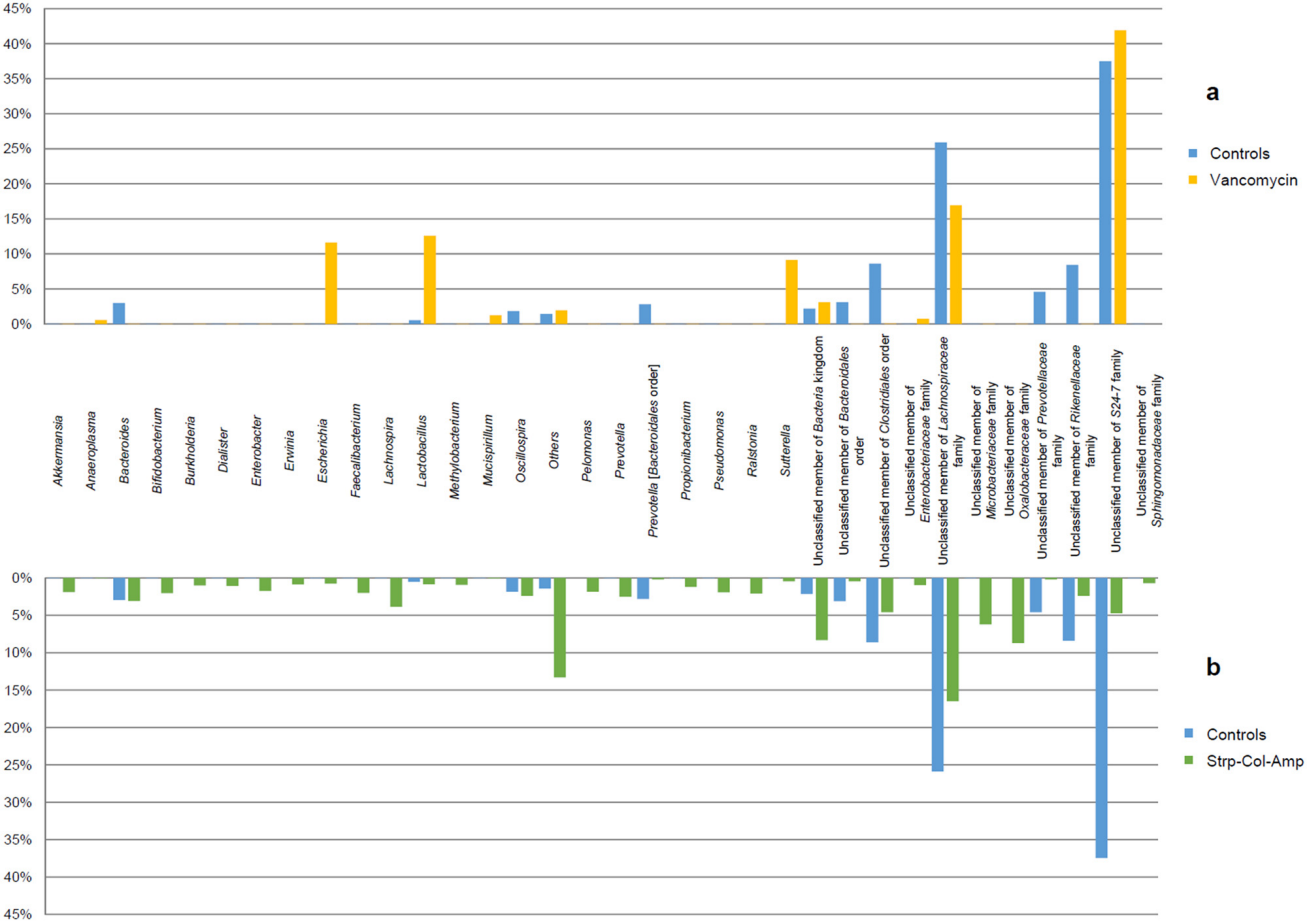
Impact of the antibiotic treatment on microbiota composition of mice at genus level. The two bar plots show the variation of bacterial taxa at genus level when mice are treated with vancomycin **(a)** and Strp-Col-Amp **(b)**. Only taxa with relative abundance in at least one sample > 2% and with a variation > 0.1% are shown.

Vancomycin treatment resulted in increased *Escherichia*, *Lactobacillus* and *Sutterella* genera and in decreased members of the *Clostridiales* order and *Lachnospiraceae*, *Prevotellaceae* and *Rikenellaceae* families, as compared to control mice (Fig 4a). Furthermore, Strp-Col-Amp treatment had an even higher impact on the microbiota, causing a strong reduction of members of the *S24-7* family, as well as reduction of the *Escherichia* and *Lactobacillus* genera, compared to the vancomycin-treated mice (Fig 4b).

### Impact of vancomycin treatment on intestinal subsets T cell subsets and their cytokine production in NOD mice

The differentiation and homeostasis of intestinal T cells including IL-17-producing T cell subsets (i.e. CD4+TCRαβ and TCRγδ T cells) and CD4+FoxP3+ regulatory T cells (Treg) are greatly impacted by the composition of the microbiota [30–32]. We therefore analyzed the proportion of these various cell subsets in the lamina propria of the ileum and colon of vancomycin-treated mice as compared to controls. Spleen cells were also analyzed.

**Fig 5:**
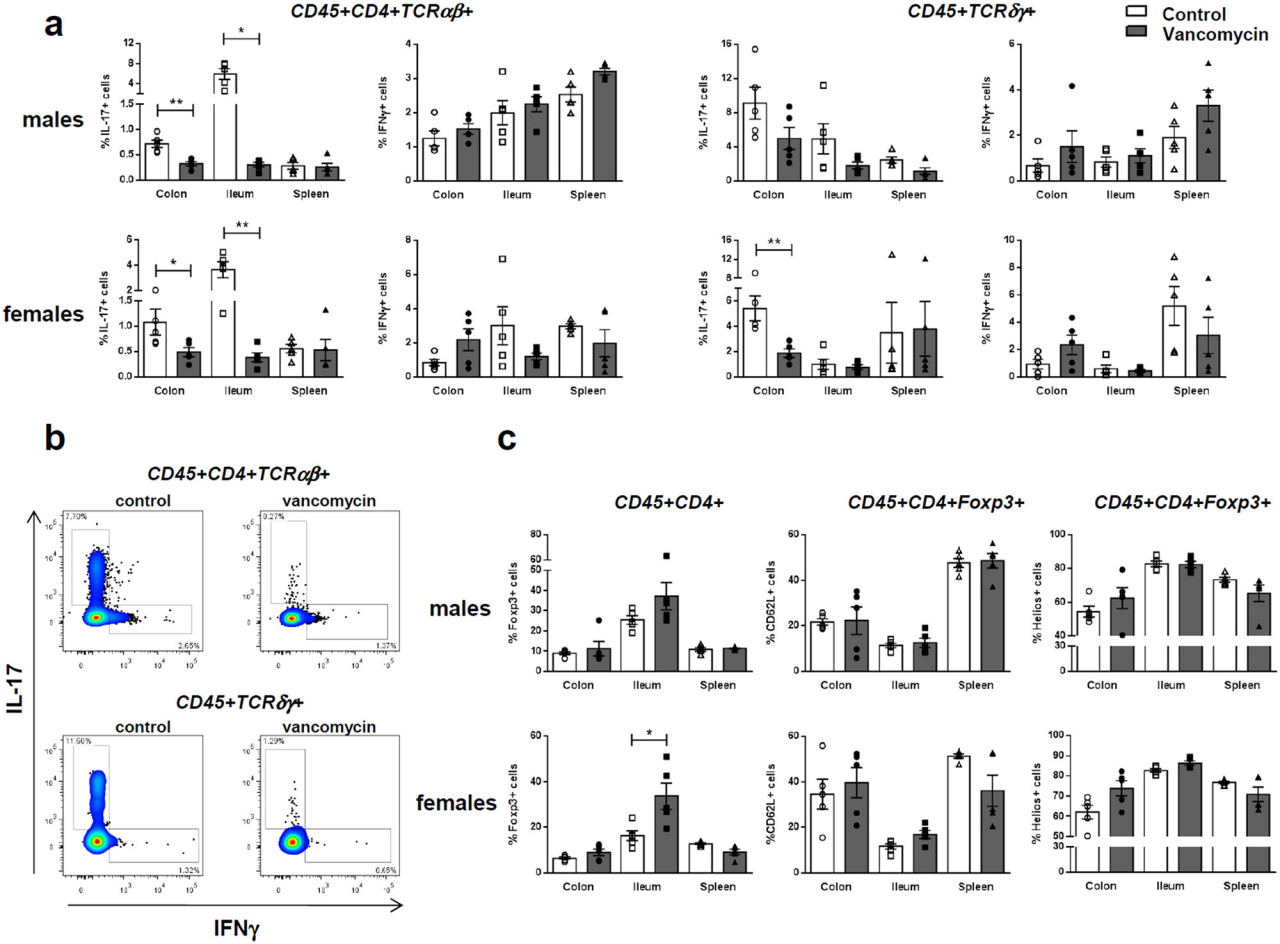
Immune cells in the intestinal lamina propria and spleen. **(a)** Percentages of IFNγ and IL-17-producing T cells (upon 6-hr stimulation with PMA/ionomycin) among CD45+CD4+TCRαβ+ and CD45+TCRγδ+ T cells isolated from the spleen and the lamina propria of the colon and ileum of 10 week-old vancomycin-treated mice (black bars) *versus* controls (open bars). The data in males (upper panels) and in females (lower panels) are shown. Five animals per group were analyzed. Significant differences are indicated as follows: * p<0.05; ** p<0.01. **(b)** Representative dot plots of intracytoplasmic staining to detect IFNγ and IL-17-producing T cells among CD45+CD4+TCRαβ+ or CD45+TCRγδ+ T cells. **(c)** Percentages of Foxp3+ cells among CD4+CD45+ T cells and of CD62L+ cells and Helios+ cells among CD45+CD4+FoxP3+ lymphocytes in 10-week-old NOD males (upper panels) and females (lower panels) treated with vancomycin (black bars) or controls (open bars). Significant differences are indicated as follows: * p<0.05; ** p<0.01.

A major decrease in the proportions of IL-17-producing CD4+TCRαβ+ cells was observed in the colon and ileum of both vancomycin-treated NOD males and females (i.e. in the colon p = 0.0079 and p = 0.0465 in males and females respectively; in the ileum p = 0.0119 and p = 0.0079 in males and females respectively, Fig 5a). A decrease in IL-17-producing TCRγδ+ T cells was also observed in the colon of vancomycin-treated males and females that was however significant only in females (p = 0.0079). A tendency towards a decrease was also observed in the ileum of treated males (Fig 5a). No differences were observed in these same IL-17-producing lymphocyte subsets in the spleen. Similar proportions of IFNγ-producing CD4+TCRαβ+ or TCRγδ+ T cells were found in the colon, ileum and spleen of vancomycin-treated *versus* control mice.

Results shown in Fig 5c revealed an increase in the proportions of FoxP3+ cells among CD45+CD4+ lymphocytes in the ileum of vancomycin-treated mice that was significant in females (p = 0.0159) but not in males (p = 0.0952).

**Fig 6:**
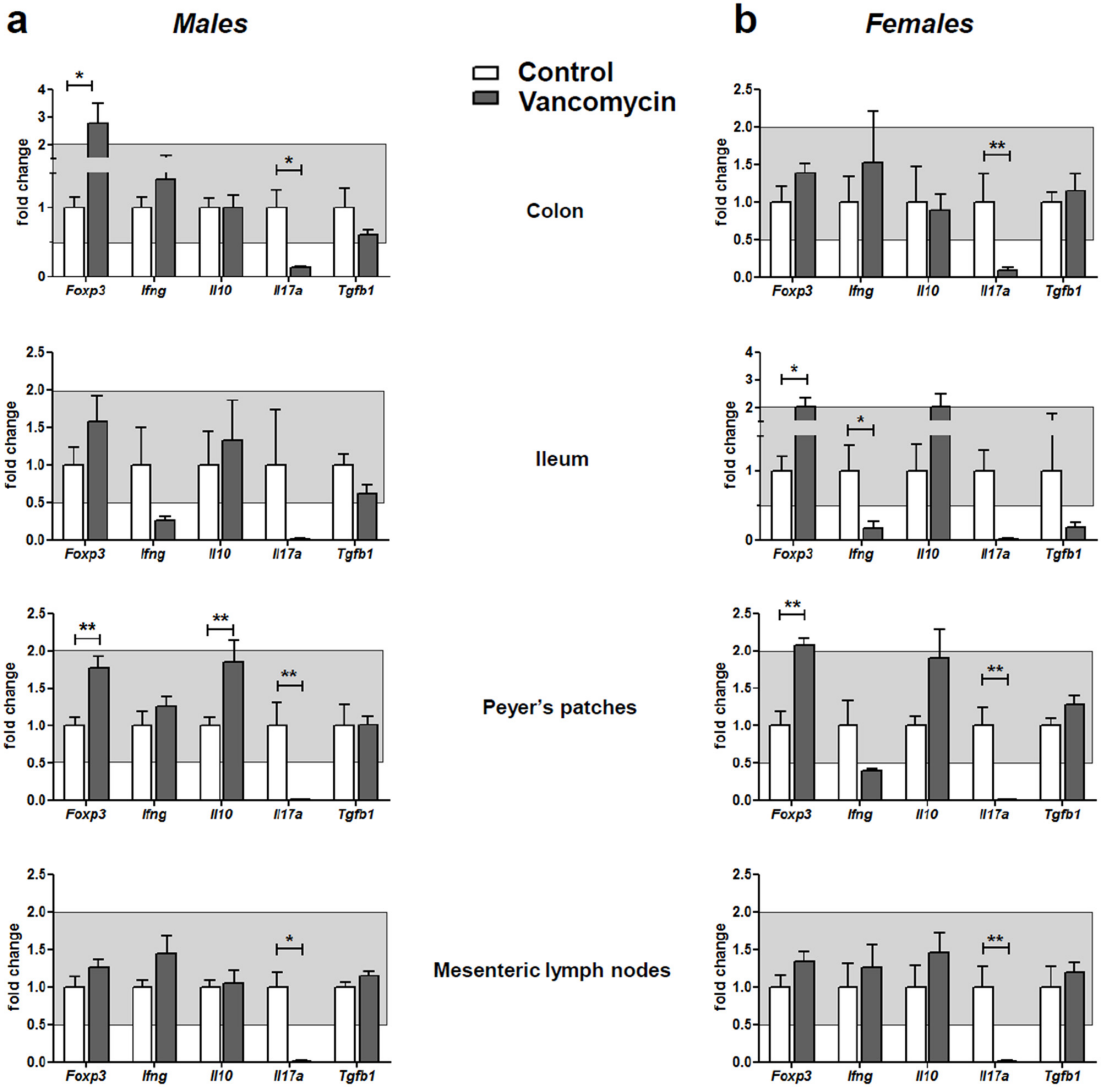
Analysis of gene expression in the gut. Relative expression of *Foxp3*, *Ifng*, *Il10*, *Il17a* and *Tgfb1* in the colon, ileum, Peyer’s patches and mesenteric lymph nodes of 10-week-old male **(a)** and female **(b)** mice treated with vancomycin (grey bars) and controls (open bars). Five animals per group were analyzed. Expression of *Foxp3*, *Ifng*, *Il17a* and *Il10* was normalized to that of *Cd3*, while expression of *Tgfb1* was normalized to *Hprt* expression. Results are expressed as fold of the mean expression observed in untreated mice. Only changes in expression levels outside the grey zone indicates changes in expression >2-fold increase or decrease in vancomycin-treated mice (black bars) as compared to controls (open bars). Significant differences are indicated as follows: * p<0.05; ** p<0.01.

The expression of the following genes was analyzed by quantitative PCR in the colon, ileum, Peyer’s patches and mesenteric lymph nodes: *Foxp3*, *Ifng*, *Il10*, *Il17a* and *Tgfb1* (Fig 6a and 6b). The salient result was a major reduction in the expression of *Il17a* in the colon, the ileum, Peyer’s patches and mesenteric lymph nodes of male and female vancomycin-treated mice (all differences were statistically significant except those in the ileum that were borderline (p = 0.0635 in males and p = 0.0593 in females)). Reduced expression of *Ifng* was observed only in the ileum of treated mice; the difference was statistically significant only in females. An increase in the expression of *Foxp3* was found in vancomycin-treated mice as compared to untreated mice. This increase was statistically significant in the Peyer’s patches of both male and female treated mice, in the ileum of treated females and the in colon of treated males. An increase in *Il10* expression was observed in Peyer’s patches of treated mice which, however, was significant only in males.

## Discussion

The observations reported here show that antibiotic treatment applied to NOD mice early in ontogeny, during fetal and postnatal life, increases the incidence of diabetes.

Three complementary elements must be highlighted:

1. the effect is observed both with a mixture of antibiotics (streptomycin, colistin and ampicillin), which virtually eradicates the whole gut microbiota but also with a single antibiotic, vancomycin, which eliminates only part of gut commensals,
2. the increase in the incidence of diabetes parallels a disappearance of IL-17-producing cells in the intestinal lamina propria and,
3. the effect is only observed in male mice.

Vancomycin has been used in many experimental models to study the effect of a significant yet partial ablation of the microbiota on the immune system [7,8,33,34]. Using this antibiotic the group of D. Littmann showed that intestinal immune cells lost their ability to produce IL-17 [34], which then led to the discovery of the selective role of the segmented filamentous bacterium (SFB) on the differentiation of Th17 cells [30,35]. More recently, the group of B. Finley reported that treatment of pregnant mice and their offspring with vancomycin until the age when immunization with ovalbumin was performed, resulted in a major increase in the allergic asthmatic response to this antigen [7]. Furthermore, it was sufficient to treat pregnant mothers and newborns until three weeks of age to have the same effect [8]. In contrast, treatment of adult mice was without effect [7]. These results are in keeping with our present ones. Conversely, they differ from those reported by Hansen et al. showing that treatment of NOD mice with vancomycin, started from birth to weaning (day 28), had no accelerating effect but instead reduced the incidence of T1D [33]. In addition, in this work *Akkermansia muciniphila* became dominant following vancomycin treatment, which we did not observed in our study. This difference in the induced intestinal dysbiosis may explain the discrepancy. Another major difference between this work and ours is that only newborn mice up to weaning were treated but not pregnant mothers. The absence of an accelerating effect of vancomycin in this condition may suggest the importance of treatment during pregnancy to impact on diabetes progression. This observation is in keeping with another recent report showing that exclusive intranasal administration of the nonpathogenic bacterium *Acinetobacter lwoffii* F78 during gestation was sufficient to mitigate allergy in the offspring [36].

Using the 16S rRNA microbial profiling, we confirmed that the antibiotics had induced profound alterations of the intestinal microbiota consistent with existing data [7,34]. Treatment with the antibiotic mixture led to an almost complete disappearance of the microbiota. In contrast, vancomycin treatment resulted in partial ablation of the microbiota primarily removing Gram positive bacteria but also impacting Gram negative ones [7,34]. Thus, as compared to controls, vancomycin-treated mice showed a decrease in members of the *Clostridiales* order and *Lachnospiraceae* family (both Gram positive bacteria from the Firmicutes phylum), as well as in the *Prevotellaceae* and *Rikenellaceae* families (Gram negative bacteria from the Bacteroidetes phylum). In addition, a compensatory increase in genera from the Proteobacteria phylum such as *Escherichia* and *Sutterella* were also observed. It is interesting that the genus *Lactobacillus* (Gram positive bacteria from the Firmicutes phylum) was proportionally increased in vancomycin-treated mice that showed an increased in the incidence of T1D. This observation may seem paradoxical considering the data in the literature describing the protective role of lactobacilli in allergic diseases and those, including our own, showing the same protective effect in autoimmune diabetes in NOD mice and BB-DP rats [21–23,37–39]. However, it is important to note that first, the intestinal genus *Lactobacillus* includes numerous species, which do not all show a protective activity in allergy and autoimmunity. An interesting example reported by R. Valladares et al. is that of *Lactobacillus reuteri* that did not protect from the development of T1D in BB rats whereas *Lactobacillus Johnsonii* 6.2 was protective [23]. Secondly, it has been well established that not all *Lactobacillus* species are similarly affected by vancomycin [40].

The Strep-Col-Amp treatment led to a situation very similar to that of GF mice in which an increased frequency of T1D has been reported [16,19]. It is tempting to suggest that the increase in the autoimmune reaction observed in this situation is related to a decrease or even the absence of Treg cells, in particular FoxP3+ cells, although this is far from proven. This is supported by the observation made by several authors that the generation or differentiation of these cells depends on certain commensal bacteria such as *Clostridium* and *Bacteroides fragilis* [41,42]. Other mechanisms can also be envisaged, particularly the stimulation of innate immunity receptors by *Toll like* receptor (TLR) ligands expressed by commensal bacteria. It is indeed striking, as we reported, that the systemic administration of TLR agonists results in complete protection from diabetes in NOD mice [21].

Results observed with vancomycin raise the question of whether certain commensal bacteria are preferentially involved in the protection against diabetes, insofar as the effect is obtained by eliminating only a portion of the microbiota. It is not possible at this stage to incriminate a precise bacterium or a group of specific bacteria, except perhaps the lactobacilli mentioned above.

As expected, the modifications of the microbiota upon vancomycin treatment greatly impacted lamina propria immune cells. Overall, an increase in either the proportions of CD4+FoxP3+ T cells or the expression of *Foxp3* mRNA was observed in the lamina propria of the ileum and colon as well as in Peyer’s patches in vancomycin-treated mice which, however, was not always significant. This is at variance with the results of Russel et al. showing that vancomycin treatment reduced CD4+CD25+FoxP3+ Treg cell accumulation in the colon [7]. Differences linked to the models used (i.e. genetic background, induced *versus* spontaneous disease) may explain the discrepancy.

More strikingly, a complete elimination of IL-17-producing cells, especially CD4+TCRαβ but also TCRγδ+ T cells, was observed in the ileum and the colon of treated mice. Analysis by quantitative PCR revealed the same important decrease in the *Il17a* gene expression in the Peyer’s patches and mesenteric lymph nodes.

At this point it is interesting to mention the major difference between T1D, where the pathophysiological role of IL-17 is still debated [43–45] as opposed to experimental autoimmune encephalomyelitis (EAE), a murine model of multiple sclerosis, where the pathogenic role of IL-17 is well established. Indeed, helper T cells producing IL-17 (Th17) play a key role in the development of EAE and, in fact, the GF status or even antibiotics that cause decreased production of IL-17 prevent the onset of EAE [46,47]. In any case, it is interesting to observe that in our model the antibiotics used, including the very broad spectrum mixture of antibiotics, which caused a near complete elimination of the microbiota, did not prevent the development/differentiation of pathogenic lymphocytes that cause autoimmune diabetes.

Of note, despite a similar impact of vancomycin on gut microbiota and lamina propria Th17 cells in male and female NOD mice, the significant increase in T1D incidence was essentially observed in males. However, both vancomycin-treated male and female mice showed a trend towards more severe islet infiltration than untreated animals at 10 weeks of age, suggesting a similar effect of vancomycin treatment on the differentiation of pathogenic lymphocytes in the two genders. The high incidence of T1D in control NOD females (>90%) may explain the apparent limited impact of vancomycin in these animals.

Our present results raise the issue of the potential triggering effect of antibiotics given to children in the first weeks of life on certain immune diseases.

We did not find epidemiological evidence to support this in T1D [48]. There is more evidence in allergic diseases. Clear results associating antibiotic intake with asthma and eczema were reported by several groups and confirmed by a meta-analysis [49–51]. Although these results are disputed by some other authors it remains that, if confirmed, the observation is alarming [52]. Is there a similar trend for autoimmune diseases, especially as according to the hygiene hypothesis a decreased exposure to infections predisposes to the increase of both autoimmune and allergic diseases?

Our present results comfort this hypothesis, particularly with regard to autoimmune diabetes and should prompt conducting prospective epidemiological trials to rigorously evaluate the risk and attempting to dissect the respective role of the different antibiotics.

## Supporting Information

S1 Table Quantitative data of the 16S rRNA gene sequence data set used in this study. (DOC)
