# Correction: Antibiotics in Early Life Alter the Gut Microbiome and Increase Disease Incidence in a Spontaneous Mouse Model of Autoimmune Insulin-Dependent Diabetes

**DOI:** 10.1371/journal.pone.0147888

**Published:** 2016-01-22

**Authors:** Sophie Candon, Alicia Perez-Arroyo, Cindy Marquet, Fabrice Valette, Anne-Perrine Foray, Benjamin Pelletier, Christian Milani, Marco Ventura, Jean-François Bach, Lucienne Chatenoud

The seventh author's name is spelled incorrectly. The correct name is: Christian Milani.
