# Supplementary material for: Antibiotics in Early Life Alter the Gut Microbiome and Increase Disease Incidence in a Spontaneous Mouse Model of Autoimmune Insulin-Dependent Diabetes
Source: PLoS One. 2015 May 13;10(5):e0125448. doi: 10.1371/journal.pone.0125448 (PMC4430542; doi:10.1371/journal.pone.0125448)
Supplement: S1 Table — (DOC) [file pone.0125448.s001.doc]

**S1 Table: Quantitative data of the 16S rRNA gene sequence datasets used in this study**

| **Sample** | **Number of reads** | **Number of reads removed because of:** | | | | | | **Final read number** | **average reads length** |
| --- | --- | --- | --- | --- | --- | --- | --- | --- | --- |
|  |  | **Outside bounds (140-400)** | **Ambiguous bases** | **Mean quality <25** | **Homopolymer runs >7bp** | **Primer mismatch >1** | **Low quality window truncation results in <140bp** |  |  |
| **1680** | 114875 | 12480 | 0 | 0 | 215 | 4750 | 2968 | 94462 | 201,3 |
| **1681** | 170688 | 20228 | 0 | 0 | 338 | 8945 | 4445 | 136732 | 201,5 |
| **1682** | 224020 | 33886 | 0 | 0 | 210 | 8718 | 5137 | 176069 | 201 |
| **1683** | 103655 | 28798 | 0 | 0 | 91 | 6912 | 2125 | 65729 | 197,6 |
| **1694** | 293087 | 42297 | 0 | 0 | 824 | 13304 | 7583 | 229079 | 198,1 |
| **1695** | 448887 | 80162 | 0 | 0 | 691 | 20116 | 12657 | 335261 | 197,3 |
| **1696** | 498582 | 81067 | 0 | 0 | 669 | 29853 | 14476 | 372517 | 198,6 |
| **1697** | 491635 | 103271 | 0 | 0 | 608 | 27011 | 16663 | 344082 | 201,9 |
| **1698** | 357103 | 62093 | 0 | 0 | 476 | 30987 | 10371 | 253176 | 194,9 |
| **1699** | 253063 | 39968 | 0 | 0 | 703 | 17570 | 8398 | 186424 | 196,7 |
| **1700** | 225856 | 31875 | 0 | 0 | 222 | 16486 | 6964 | 170309 | 201 |
| **1706** | 224312 | 42463 | 0 | 0 | 205 | 9358 | 6334 | 165952 | 200,6 |
| **25** | 104159 | 48656 | 0 | 0 | 8 | 3725 | 4664 | 47106 | 186,3 |
| **26** | 127666 | 30702 | 0 | 0 | 17 | 5581 | 6968 | 84398 | 184,8 |
| **27** | 157792 | 62832 | 0 | 0 | 64 | 6752 | 7855 | 80289 | 184,9 |
| **30** | 142766 | 39549 | 0 | 0 | 10 | 6246 | 7829 | 89132 | 184,9 |
| **32** | 158654 | 71081 | 0 | 0 | 10 | 6732 | 7978 | 72853 | 186,2 |
| **35** | 235581 | 112070 | 0 | 0 | 27 | 6777 | 9906 | 102477 | 185,2 |
| **36** | 170630 | 38853 | 0 | 0 | 21 | 14920 | 7303 | 109533 | 187,3 |
| **37** | 122476 | 87484 | 0 | 0 | 11 | 2167 | 3285 | 29529 | 172,5 |
| **38** | 115445 | 62317 | 0 | 0 | 35 | 3076 | 1323 | 48694 | 172,7 |
| **39** | 177738 | 115659 | 0 | 0 | 28 | 2929 | 1856 | 57266 | 172,9 |
| **40** | 368267 | 153346 | 0 | 0 | 68 | 13951 | 6105 | 194797 | 172,3 |
| **41** | 186592 | 149677 | 0 | 0 | 19 | 1711 | 1224 | 33961 | 172,6 |
| **43** | 149587 | 109196 | 0 | 0 | 27 | 2108 | 3021 | 35235 | 173,6 |
| **44** | 1278632 | 1271231 | 0 | 0 | 1 | 413 | 597 | 6390 | 172,5 |
| **45** | 88006 | 73937 | 0 | 0 | 14 | 1439 | 834 | 11782 | 172,5 |
| **47** | 63149 | 50994 | 0 | 0 | 8 | 922 | 680 | 10545 | 172,5 |
| **48** | 98824 | 73592 | 0 | 0 | 4 | 1662 | 1845 | 21721 | 173,0 |
| **107** | 31276 | 17690 | 0 | 0 | 51 | 561 | 1027 | 11947 | 176,3 |
| **108** | 79971 | 56725 | 0 | 0 | 55 | 1466 | 665 | 21060 | 176,8 |
| **109** | 40959 | 7840 | 0 | 0 | 56 | 1745 | 892 | 30426 | 175,6 |
| **110** | 78709 | 23828 | 0 | 0 | 60 | 2667 | 1834 | 50320 | 185,6 |
| **111** | 59223 | 11304 | 0 | 0 | 156 | 2328 | 1492 | 43943 | 184,3 |
| **112** | 110524 | 12096 | 0 | 0 | 40 | 5007 | 3157 | 90224 | 184,2 |
| **113** | 62320 | 6656 | 0 | 0 | 167 | 2759 | 1627 | 51111 | 185,6 |
|  |  |  |  |  |  |  |  |  |  |
